# Chinese Cardiovascular Disease Mobile Apps’ Information Types, Information Quality, and Interactive Functions for Self-Management: Systematic Review

**DOI:** 10.2196/mhealth.8549

**Published:** 2017-12-14

**Authors:** Bo Xie, Zhaohui Su, Wenhui Zhang, Run Cai

**Affiliations:** ^1^ School of Nursing The University of Texas at Austin Austin, TX United States; ^2^ School of Information The University of Texas at Austin Austin, TX United States; ^3^ School of Advertising and Public Relations The University of Texas at Austin Austin, TX United States; ^4^ Department of Statistics and Data Science The University of Texas at Austin Austin, TX United States; ^5^ Chongqing Cancer Institute Chongqing China

**Keywords:** mobile health, mHealth, cardiovascular disease, CVD, patient preferences, information quality, self-management, mobile applications, mobile apps, China

## Abstract

**Background:**

China has a large population with cardiovascular disease (CVD) that requires extensive self-management. Mobile health (mHealth) apps may be a useful tool for CVD self-management. Little is currently known about the types and quality of health information provided in Chinese CVD mobile apps and whether app functions are conducive to promoting CVD self-management.

**Objective:**

We undertook a systematic review to evaluate the types and quality of health information provided in Chinese CVD mobile apps and interactive app functions for promoting CVD self-management.

**Methods:**

Mobile apps targeting end users in China with CVD conditions were selected in February 2017 through a multi-stage process. Three frameworks were used to evaluate the selected apps: (1) types of health information offered were assessed using our Health Information Wants framework, which encompasses 7 types of information; (2) quality of information provided in the apps was assessed using the 11 guidelines recommended by the National Library of Medicine of the National Institutes of Health; and (3) types of interactive app functions for CVD self-management were assessed using a 15-item framework adapted from the literature, including our own prior work.

**Results:**

Of 578 apps identified, 82 were eligible for final review. Among these, information about self-care (67/82, 82%) and information specifically regarding CVD (63/82, 77%) were the most common types of information provided, while information about health care providers (22/82, 27%) and laboratory tests (5/82, 6%) were least common. The most common indicators of information quality were the revealing of apps’ providers (82/82, 100%) and purpose (82/82, 100%), while the least common quality indicators were the revealing of how apps’ information was selected (1/82, 1%) and app sponsorship (0/82, 0%). The most common interactive functions for CVD self-management were those that enabled user interaction with the app provider (57/82, 70%) and with health care providers (36/82, 44%), while the least common interactive functions were those that enabled lifestyle management (13/82, 16%) and psychological health management (6/82, 7%). None of the apps covered all 7 types of health information, all 11 indicators of information quality, or all 15 interactive functions for CVD self-management.

**Conclusions:**

Chinese CVD apps are insufficient in providing comprehensive health information, high-quality information, and interactive functions to facilitate CVD self-management. End users should exercise caution when using existing apps. Health care professionals and app developers should collaborate to better understand end users’ preferences and follow evidence-based guidelines to develop mHealth apps conducive to CVD self-management.

## Introduction

Cardiovascular disease (CVD) is a leading cause of morbidity and mortality in China, accounting for 45% of all deaths in rural areas and 42% in urban areas [1]. Currently, approximately 290 million people in China live with CVD [2]. Meanwhile, there is a severe shortage of doctors in China, where the doctor-patient ratio is 1.4 doctors per 1000 patients [3]. Mobile health (mHealth) apps therefore hold promise for delivering health information and services to Chinese patients, especially for chronic conditions like CVD, which require extensive self-management [4]. Self-management is key to person-centered care [5], but its support requires an understanding of individual preferences for different types of health information and decision-making autonomy [6]. The Health Information Wants (HIW) framework suggests 7 types of information that patients typically desire in health care contexts: (1) information about the specific health condition, (2) treatment, (3) laboratory tests, (4) self-care, (5) complementary and alternative medicine (CAM), (6) psychosocial aspects, and (7) health care providers (HCPs) [6-8]. Empirical research using the HIW framework has shown that diabetes-related mobile apps for Mainland Chinese users offer inadequate information [9]. However, little is currently known about whether Chinese CVD mobile apps might offer a broad range of high-quality information and functions that can facilitate effective CVD self-management.

The self-management of chronic conditions requires the ability “to manage the symptoms, treatment, physical and psychosocial consequences and life style changes inherent in living with a chronic condition” [10]. Self-management is inherent to person-centered care that promotes a “balanced consideration of the values, needs, expectations, preferences, capacities, and health and well-being of all the constituents and stakeholders of the health care system” [5]. Effective self-management and person-centered care require full accommodation of people’s needs and preferences for different types and amounts of information and other care services [11,12], a degree of autonomy in health-related decision-making [13,14], and support from their formal (eg, health care professionals) and informal caregivers (eg, family members) [15,16].

Although mHealth apps have the potential to promote effective self-management and person-centered care [17,18], existing apps tend to provide limited types of information [9], lack information quality assurance [19-21], and offer inadequate functionalities [22]. These limitations may have negative impacts on mHealth app users. Information quality is important to users of electronic health (eHealth) and mHealth products or services [23,24]. However, the quality of online health information is often unregulated and problematic [25], especially in the context of mHealth apps [23,24]. The situation is even worse in developing countries, including China [26-28]. One study, for example, has found that the quality of Chinese mHealth apps is poor, especially due to limited coverage of medication topics [29]. Another study found that Chinese mHealth apps tend to lack information accountability and have limited coverage of relevant topics [30]. Empirical evidence also suggests that concerns about privacy and information quality could hinder Chinese people’s mHealth app usage [31].

Nevertheless, mobile apps’ interactive functions have the potential to assist users in their health care activities and improve user satisfaction [32-34]. A randomized controlled trial undertaken in Sweden, for example, found that an interactive health app was more effective than a noninteractive tool for improving medication adherence, lifestyle changes, and quality of life [35]. However, little is currently known about the types of interactive functions in Chinese CVD apps and whether they might meet existing guidelines for promoting CVD self-management; even studies that have focused on Chinese CVD-related apps have not considered these characteristics [21,30].

In the present study, we assessed (1) the types of health information that Chinese CVD apps offer, (2) the quality of information available in the apps, and (3) the apps’ interactive functions for promoting CVD self-management.

## Methods

We selected the mobile apps for this study in multiple steps. First, in February 2017, we conducted searches on the Chinese website for the iTunes App Store, using predetermined search terms consistent with New York State Department of Health definitions of CVD [36] and based on our research team’s knowledge of the terms that Chinese people typically use when describing CVD-related conditions (one of our research team members, a surgeon, specializes in CVD-related conditions and has practiced in China for over 20 years). The use of lay people’s search terms enabled us to obtain the types of apps that people in China would typically find in their own searches (see Textbox 1).

The initial search yielded 578 results. After we removed duplicates, 464 apps remained. Next, we reviewed the titles and descriptions of the remaining apps in iTunes and included those that: (1) targeted Mainland China; (2) had full text in simplified Chinese characters (used in Mainland China); (3) targeted end users/patients/the general public; and (4) covered at least one CVD condition. Apps that did not meet all four of these criteria were excluded. A total of 98 apps remained in the review.

**Figure 1:**
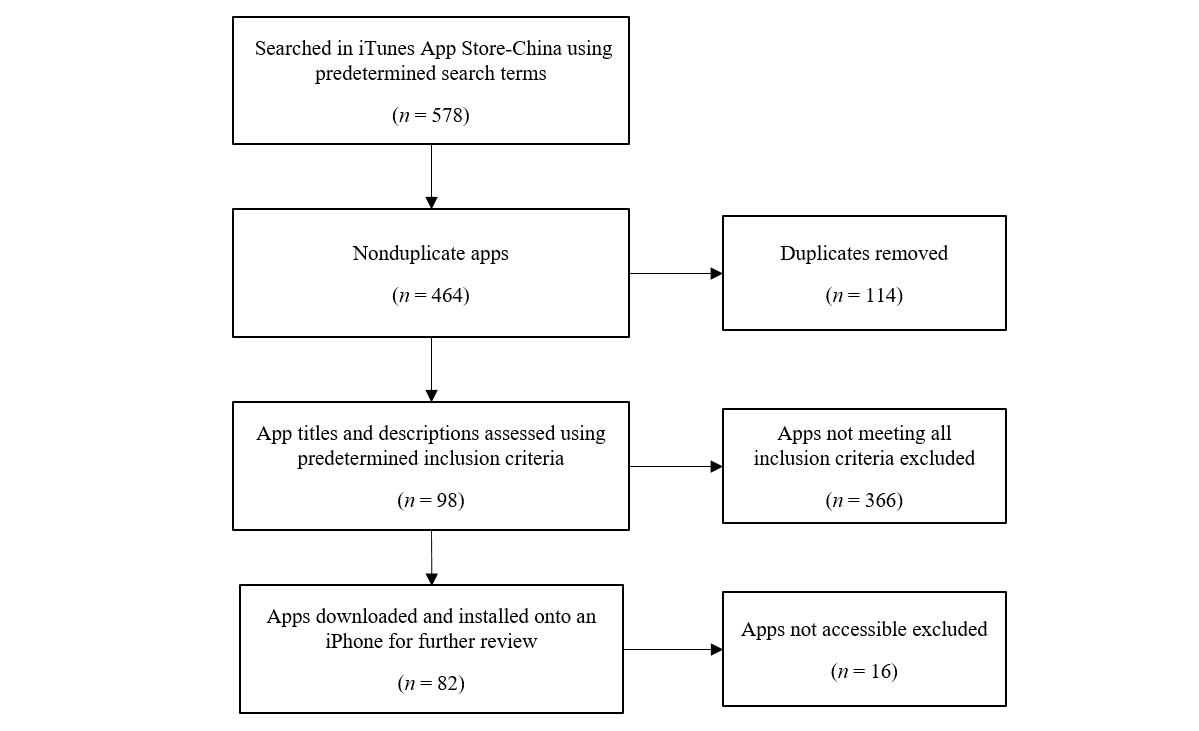
App selection process.

Finally, we downloaded (or attempted to download) each of the 98 apps to an iPhone for further examination. During this step, we found that 16 of the apps were not accessible or downloadable, so they too were excluded. The final sample consisted of 82 apps that could be downloaded and installed for further examination (see Figure 1).

**Chinese search terms used.**

心脏病 (heart diseases)

心脑血管 (cardiovascular diseases)

中风 (stroke)

卒中 (stroke)

冠心病 (coronary artery disease)

高血压 (high blood pressure)

偏瘫 (paralysis)

After downloading and installing the 82 apps onto an iPhone to simulate Chinese end users’ experiences, we used the HIW framework, the National Library of Medicine’s (NLM’s) guidelines, and our own self-management framework to systematically evaluate the apps’ (1) information types, (2) information quality, and (3) interactive functions for self-management, respectively. One researcher (a trained graduate research assistant) first coded five apps using these frameworks. The coding results were verified by another researcher (a faculty member experienced with this type of research). Differences between these two researchers were resolved through discussions. The research assistant then completed the coding for all apps in the final sample, with the faculty member randomly verifying 10% of the results; differences were again resolved through discussions. The research assistant then revised relevant coding based on the discussions.

### The Health Information Wants Framework for Evaluating the Types of Information

We adapted the HIW framework [37] to assess the types of health information provided in the apps. As stated in the introduction, this framework covers 7 types of health information commonly wanted by patients in health care encounters; based on the descriptions for each type and subtype from our earlier studies [9,37], we adapted items to apply to CVD specifically, when necessary (eg, *information about the type of diabetes* was changed to *information about the type of CVD*). The presence of each type of health information was recorded as 1, and each absence was recorded as 0 (scoring range: 0-7). The higher the score, the more types of information identified in the apps.

### The National Library of Medicine Framework for Evaluating Quality of Information

Based on the NLM guidelines [38], we developed operational definitions to determine the quality of the apps’ information. This framework includes 11 indicators of information quality; the presence of each indicator was recorded as 1, and each absence was recorded as 0 (scoring range: 0-11). The higher the score, the higher the app’s information quality (Table 1).

### The Self-Management Framework for Evaluating Interactive App Functions

A major advantage of mobile apps is that they enable user input (eg, recording of physical activities) and interaction with others (eg, HCPs, families, and peers) that are key to effective self-management. Current app evaluation frameworks tend to focus on limited interactive functions. For instance, the Mobile Apps Review Scale [39] narrowly defines interactivity within the parameters of user input, feedback, and prompts, with no consideration of other important interactive functions (eg, virtual rewards, online-offline integration, family involvement). Hale et al [40] developed an evaluation framework to assess the congruency between apps’ intervention strategies and user needs. However, their framework was designed to assess apps’ topics (eg, healthy eating) and intervention strategies (eg, self-monitoring); it did not offer a systematic way to evaluate the scope of interactive app functions (the authors mention only briefly that self-monitoring, social support, modelling/vicarious learning, and stimulus control are highly interactive strategies that an app can adopt) [40].

Based on the literature on strategies for effective self-management of chronic conditions [4,6,7,9,10,41-43] as well as the aforementioned frameworks for evaluating mobile apps [39,40], we developed our 15-item Self-Management Framework for Evaluating Interactive App Functions (SFEIAF) to specifically assess interactive functions that are necessary for effective CVD self-management in CVD apps. We did not, however, include *providing information* as an app function in our SFEIAF, even though providing relevant information to ensure patient education is essential for effective self-management. This decision was based on two reasons: (1) the function of providing patient education information is already fully covered in our HIW framework, which focuses specifically on a broad range of health information; and (2) the typical approach to providing patient education information is top-down, such that information flows from the app developer to the user with no input from the user. In contrast, our SFEIAF emphasizes user input in information provision that is essential to effective self-management. The presence of each function in the SFEIAF was recorded as 1, and each absence was recorded as 0 (scoring range: 0-15). The higher the score, the more the interactive functions in an app (Table 2).

**Table 1:** National Library of Medicine guidelines for information quality and our operational definitions.

NLM guideline	Operational definitions
Providing information on who is in charge of the app	Provides information that could help users understand who is in charge of the app (eg, information about the app provider’s name). Such information is typically found via the *About Us* button in the app and/or on the app’s iTunes page.
Providing information about why the app is being provided	Provides information that could help users understand the app’s purpose, intended users, and functionalities. Such information is typically found on the app’s iTunes page and/or via the *About Us* button in the app (eg, indicating that the app is developed for CVD^a^ patients, or to provide CVD-related information, or to provide one-on-one consultation with a CVD physician).
Providing the app provider’s physical address	Provides information about the physical address of app developer or administrator.
Providing information on the source of the app’s information	Provides information that could help users understand where the information used by the app came from (eg, an article or book with author names, or, for Web-based information, the website from which the information was retrieved).
Providing information on how the app’s content was selected	Provides a logical explanation for how the app’s information was selected (eg, information selected from peer-reviewed journals).
Having expert review of the information	Provides information to make clear that information presented in the app has been reviewed by qualified health care professionals.
Financial disclosure	Provides information on where the money to support an app comes from (eg, government agencies, nonprofit organizations, drug companies). This information could help users understand whether the app’s providers have financial motives that users should be aware of (eg, the sale of CVD drugs).
Content is up-to-date	The original NLM^b^ guidelines did not specify what timeframe would be considered *up-to-date*; in our study, we operationalized this indicator as app content updated in the past 3 months.
Does not have advertisements	Whether or not an app contains advertisements. Note: if a drug or treatment option mentioned in an app was a part of scientific results (eg, if reported in a research article) then it was *not* considered an advertisement.
Does not use unbelievable or emotional claims	Whether or not an app makes claims that are too good to be true, or are based on emotions instead of scientific facts (eg, “Lose 30 pounds in 2 weeks!”).
Does not ask for personal information	Whether or not users must submit personal information (eg, name, phone number, email address) in order to use certain app functions.

^a^CVD: cardiovascular disease.

^b^NLM: National Library of Medicine.

**Table 2:** Interactive app functions in the Self-Management Framework for Evaluating Interactive App Functions and their operational definitions.

Interactive app function	Our operational definition
Monitoring of physical or health indicators	Functions that allow users to record and monitor their health or physical indicators (eg, body mass index, blood pressure)
Exercise and physical activity management	Functions that enable users to record and monitor their exercise and physical activities (eg, interactive pedometers)
Lifestyle management	Functions that enable users to record and monitor aspects of their lifestyles relevant to the prevention of, or coping with, CVD^a^ (eg, monitoring of alcohol drinking)
Medication management	Functions that enable users to manage their medication (eg, when to take what medicine as prescribed)
Interaction with health care providers	Functions that enable the user to interact with HCPs^b^ (eg, consultation via the app)
Condition management or prevention	Functions (other than medication management) that enable user input or interaction to control or prevent CVD (eg, hypertension self-detector)
Psychological health management	Functions that enable users to understand or manage their psychological health (eg, self-evaluation of psychological health)
Peer interaction	Functions that enable users to interact with other users with similar health conditions (eg, in-app peer support groups)
Family involvement	Functions that enable users to include family in their CVD self-management
Virtual rewards/gamification	Functions that provide motivational or gamification functions to encourage user commitment to their CVD self-management (eg, virtual rewards to encourage medication adherence)
Personal health records management	Functions that enable user input of their health-related data (eg, electronic health profiles)
Individualized care management	Functions that enable tailoring of prompts based on user input (eg, care recommendations tailored to users’ specific health conditions)
Multiple platform care management	Functions that enable users to connect their CVD care concerns across multiple platforms (eg, short message reminders sent from the app to other apps or electronic services such as email)
Online-offline integration	Functions that enable users to connect and integrate their online and offline self-management activities (eg, connecting the app with a blood glucose device to manage one’s blood sugar level)
Interaction with the app provider	Functions that enable users to communicate with, and receive feedback from, the app provider. Depending on the questions that users ask, this type of interaction could involve technical support (eg, questions about how to use the app) or medical issues (eg, CVD-specific questions).

^a^CVD: cardiovascular disease.

^b^HPCs: health care providers.

## Results

### Types of Information

Ten of the 82 (10/82, 12%) apps in our final sample received scores of zero, because they offered none of the 7 types of information in the HIW framework; 15 (15/82, 18%) received scores of 1-2 (ie, they offered 1 or 2 types of information); 27 (27/82, 33%) offered 3 or 4 types; and 30 (30/82, 37%) offered 5 or 6 types of information. No app offered all 7 types of information. Self-care was the most commonly offered type of information (67/82, 82%), followed by information about the specific health condition (63/82, 77%) and treatment (55/82, 67%); information about laboratory tests (5/82, 6%) was the least common (Table 3).

### Quality of Information

Nineteen (19/82, 23%) of the apps received scores of 2-3 (ie, they met 2 or 3 NLM guidelines for information quality). Sixty-two (62/82, 75%) met 4-6 guidelines, and only one app (1/82, 1%) met 8; no app met more than that. The most commonly-met NLM guidelines were those that addressed providing information about the apps’ developers (82/82, 100%) and purpose (82/82, 100%). The least commonly-met guideline was that which addressed disclosing the app’s source of financial support; none of the apps did this (Table 4).

### Interactive App Functions for Self-Management

Nine (9/82, 11%) apps scored zero on the interactive app functions for self-management (ie, they offered none of the 15 interactive functions for CVD self-management specified in the SFEIAF). Twenty-nine apps (29/82, 35%) scored 1-3, offering 1-3 functions; 21 (21/82, 26%) offered 4-6, 12 (12/82, 15%) offered 7-9; 7 (7/82, 9%) offered 10-12; and only 4 (4/82, 5%) offered 13 functions. No app offered more than 13 functions. The most commonly offered interactive function was interaction with the app provider (57/82, 70%), and the least commonly offered was psychological health management (6/82, 7%; Table 5).

**Table 3:** Types of health information covered by Chinese CVD apps.

Type of health information	n (%)
Self-care	67 (82)
Health-condition specific	63 (77)
Treatment	55 (67)
Complementary and alternative medicine	34 (42)
Psychosocial aspects	29 (35)
Health care providers	22 (27)
Laboratory tests	5 (6)

**Table 4:** National Library of Medicine’s information quality indicators covered by the apps.

Information quality indicator	n (%)
Provided information on who is in charge of the app	82 (100)
Provided information about why the app is being provided	82 (100)
Does not use unrealizable, emotional, or sensational language	67 (82)
Does not have advertisements	46 (56)
App content is up-to-date	35 (43)
Does not ask for personal information	30 (37)
Provided information on the source of the app’s information	5 (6)
Provided the app provider’s physical address	3 (4)
Expert review of the information selected in the app	3 (4)
Provided information on how the app’s content was selected	1 (1)
Financial support disclosure	0 (0)

**Table 5:** Interactive app functions for self-management.

Interactive app function	n (%)
Interaction with the app provider	57 (70)
Interaction with health care providers	36 (44)
Online-offline integration	34 (42)
Multiple platform care management	33 (40)
Monitoring of physical or health indicator	28 (34)
Personal health records management	28 (34)
Condition management or prevention	27 (33)
Individualized care management	26 (32)
Family involvement^a^	20 (24)
Peer interaction	19 (23)
Virtual rewards/gamification	19 (23)
Medication management	18 (22)
Exercise and physical activity management	15 (18)
Lifestyle management	13 (16)
Psychological health management	6 (7)

^a^Family involvement included app features that enabled user-designated family members to access user information in the app and allowed app developers to directly send family members information (eg, general health education information related to users’ health conditions, reminders of important things for users to do, and emergency alerts).

**Table 6:** App purposes.

Category	Definition	n (%)^a^
Health education	App has a function that aims to provide information and resources that could inform people about CVD^b^ conditions	44 (64)
Self-management	App has a function that aims to help users’ own monitoring and management of their health	36 (52)
CVD risk evaluation	App has a function that aims to provide a calculator for users to assess their odds of developing one or more CVD conditions	13 (19)
Interaction with health care providers	App has a function that aims to facilitate user-health care provider communications, including making appointments and having face-to-face or virtual one-on-one medical consultations	35 (51)
Interaction with peers	App has a function that aims to allow users to communicate and bond with other users with similar health conditions	9 (13)
Family involvement	App has a function that aims to involve users’ family members in the care management process	12 (17)
Selling products and services	App has a function that aims to sell products (eg, medications) and services (eg, housekeeping services) to users	7 (10)

^a^We coded an app into multiple categories if it had multiple purposes, so the total percentage exceeds 100% (27 of the apps, or 39%, were coded as having a single purpose; the remaining 42 apps each had multiple purposes).

^b^CVD: cardiovascular disease.

### Follow-Up Analysis Results

In September 2017, following reviewers’ feedback, we performed follow-up analyses on app purposes. The definitions and results are reported in Table 6. At this point, 13 of the 82 original apps were no longer available; thus, these analyses were based on a sample of 69 apps.

In October 2017, we further examined the types of providers in charge of the apps in our final sample. Of the 69 apps that we had examined in the previous month, only 59 were still available. Of these, 48 (48/59, 81%) were developed by for-profit companies (46 by information technology/software companies; 2 by pharmaceutical companies), and 11 (11/59, 19%) were developed by individuals with no clear connection to any organization. No app in this sample was developed by government agencies or nonprofit organizations. We also analyzed the type of CVD on which the apps focused: 20 (20/59, 34%) of the apps focused on general heart health monitoring/management; 17 (17/59, 29%) on hypertension; 9 (9/59, 15%) on stroke; 6 (6/59, 10%) on diabetes; 6 (6/59, 10%) on coronary heart disease; and 1 (1/59, 2%) on heart failure.

## Discussion

Self-management is key to person-centered care, which acknowledges the importance of respecting people’s health care preferences for types and amounts of health information and social interactions (eg, family involvement in care) in their own health or disease management [9-11,13]. Effective self-management requires the building of a safe and shared care environment that can meet people’s information wants, their desires to make their own decisions, and their desires for high-quality health care and social interactions [14-16,25].

mHealth apps have the potential to assist self-management of many health conditions [17,18]. However, to ensure that mobile apps can adequately meet the wide range of users’ information wants [9,12], needs for high-quality information and care access [19,20], and self-management decision-making, evidence-based insights are needed to bridge current knowledge gaps [14,16,22]. Such insights could guide research and practice in China. In the Chinese health app market, the number of technology and health app users is growing exponentially [44,45], yet the quality of Chinese mHealth apps appears to be inferior to that of those in developed countries [27-29], and research on the current development of Chinese CVD apps has so far been limited [29,30].

In this study, we have systematically assessed (1) the types of health information that Chinese CVD apps offer, (2) the quality of health information available in the apps, and (3) the apps’ interactive functions for promoting self-management. The results show that none of the reviewed apps offered all 7 types of information specified in the HIW framework, met all 11 NLM guidelines for information quality, or provided all 15 of the SFEIAF’s interactive functions for promoting CVD self-management. Given that CVD self-management requires a variety of information [46], a lack of comprehensive information may hinder CVD self-management. For example, of all apps that were reviewed, only 5 offered information about laboratory tests, yet such information is crucial for patients [47].

None of the apps revealed sources of financial support or sponsorship, and very few revealed information about the selection of the apps’ content, app providers’ physical addresses, or the sources of the apps’ content. Over 60% of the apps asked for users’ personal information without a clear indication of how such information would be stored or used. Privacy concerns might affect app usage [48], so Chinese health app developers should integrate privacy protection measures into their future app designs. Overall, Chinese CVD apps fail to provide sufficient information for users to evaluate apps’ information quality.

Nine of the CVD apps offered none of the 15 interactive functions for self-management. This finding is troublesome, because a major advantage of mHealth apps is their ability to facilitate user input in managing their own health care [49,50]. This finding for Chinese apps, along with similar findings reported in studies of English-language mHealth apps [51,52], suggests that the same challenge exists across national/cultural boundaries. No app in our study offered more than 13 interactive functions for CVD self-management. Furthermore, although psychological health is closely associated with CVD conditions [53], only 7% (6/82) of the apps offered interactive functions that might facilitate self-management of psychological health. Given that individuals with CVD conditions or concerns may have to deal with comorbid psychological stress or other mental discomfort [54,55], it is important that this feature is addressed in future app design as well.

Facilitating family involvement is important in chronic disease self-management [56], especially in Chinese society, where the patient’s family traditionally plays a major role [57]. In China, family involvement is often considered integral to, and indispensable for, individuals’ health care [58,59]. A lack of functionality for family involvement might disconnect app users’ online self-management from the offline family-centered management of their condition. However, only 24% of the apps in our study had functions that enabled family involvement. Future app development should strive to include functions for family involvement, and should investigate and compare the efficacy of different interactive functions for family involvement to provide more guidance for app development (some interactive functions might work better for some users and their families, whereas others might find other functions more useful).

### Limitations and Future Directions

This was a cross-sectional study. Although this study is novel in that it provides, to our knowledge, the first systematic evaluation of Chinese CVD apps’ information types, information quality, and interactive functions, it does so for a snapshot of apps available when the search was performed. Future research could examine issues at multiple time points to reveal the evolution of app development. This study only examined apps developed for iOS users in Mainland China (due to limited resources, we focused only on iOS apps available in Apple’s App Store); thus, the findings should not be generalized without caution. However, the overall findings of this study do resemble those reported in a Western context and across mobile platforms [25], suggesting that such problems may be common across different cultural/national and mobile platform contexts. Future research can apply the frameworks used in this study to examine apps in other national contexts to provide more systematic comparisons across the globe. It would also be desirable to secure research funding for researchers to purchase other types of mobile devices (eg, Android) to study mobile apps running on platforms other than iOS (it is certainly not ideal to rely completely on personal devices for data collection, as we did in this study).

### Conclusions

Chinese CVD apps currently provide a limited range of high-quality information and lack sufficient interactive functions conducive to effective CVD self-management. Although family involvement in health care is expected in Chinese society, functionality for family involvement has not been adequately integrated into Chinese CVD apps. This study’s findings call for the development of more evidence-based, user-centered mHealth apps, with further systematic examination and monitoring of the apps’ abilities to provide a broad range of high-quality information and more interactive app functions to facilitate self-management of CVD.

## Abbreviations

CAM: complementary and alternative medicine
CVD: cardiovascular disease
eHealth: electronic health
HCP: health care provider
HIW: Health Information Wants
mHealth: mobile health
NLM: National Library of Medicine
SFEIAF: Self-Management Framework for Evaluating Interactive App Functions
